# Anatomy of the Arteries of the Human Body, with Descriptive Anatomy of the Heart

**Published:** 1861-05

**Authors:** 


ART. VII.—*Anatomy of the Arteries of the Human Body, Descriptive and Surgical, with the Descriptive Anatomy of the Heart.* By JOHN HATCH POWER, M.D., etc. 12mo., pp. 374. Fannin & Co.: Dublin, 1860.

THE work before us is based upon the late Dr. Valentine Flood's "Surgical Anatomy of the Arteries," published in 1839, a second edition of which was issued eleven years later, by Dr. Power, who made many important additions and alterations in the original. These additions included the anatomy of the heart, of the innominate and subclavian arteries, and the operations performed upon them, of the axilla and axillary artery; the operations on the brachial artery, the iliacs and their branches, and the femoral; the circulation of the blood in the liver and in the kidneys; and, lastly, the treatment of brachial and popliteal aneurism, by compression between the tumor and the heart. The main differences between the present edition and that of 1850, are that the former is increased in size, although the letter-press is not very materially changed, that it is abundantly and judiciously illustrated by wood-cuts, copied, or modified, from Maclise, Quain, Tiedemann, and Cloquet, and that a section has been added on the principal varieties of the arteries. Being thus enlarged and improved, without being voluminous, it presents the appearance of a new work, and will be found very valuable to the student and practitioner of medicine, as a book of reference. It deserves, and, we trust, will meet with a cordial appreciation.

In looking over the statistics of the ligature of the principal arterial trunks, we are struck with the want of more copious references, a point very desirable to those who wish to find the details of any case. Thus, under the head of ligation of the innominate, Norman, of Bath, is said to have performed the operation, in 1824, but we are not informed where the case is published, nor have we ever been able to discover. In the same table is included the operation of Dr. Cooper, of San Francisco, thus making the tenth fatal case. The same surgeon has recently performed a similar operation, the subject perishing of secondary hemorrhage, on the forty-first day. Eleven fatal cases do not, therefore, offer any special inducements for a repetition of the procedure.

Under the head of ligature of the abdominal aorta, we find the particulars of the fifth and last case, that of Mr. South, in 1856. No authentic report of this operation having been published, the following facts, communicated to Dr. Power, will prove interesting:—

"The man was thirty years of age, and a hard drinker; had had a strange, uneasy sensation two months before his admission, and six weeks after noticed a small, hard, pulsating swelling in his right groin, which grew rapidly, and when admitted was as big as a goose-egg. Soon suffered paroxysms of violent pain, and leg became numb. Eleven days after, the aorta was tied without difficulty by a cut from the tip of the tenth rib to the superior iliac spine. In the course of a few hours, first one, and subsequently the other limb became discolored; was in constant profuse perspiration, and exceedingly restless. Died forty-two hours after. Examination showed aneurism of right external iliac artery."

